# Development of Growth Charts of Pakistani Children Aged 4-15 Years Using Quantile Regression: A Cross-sectional Study

**DOI:** 10.7759/cureus.2138

**Published:** 2018-02-02

**Authors:** Sundus Iftikhar, Nazeer Khan, Junaid S Siddiqui, Naila Baig-Ansari

**Affiliations:** 1 The Indus Hospital Research Center, The Indus Hospital; 2 Department of Research, Jinnah Sindh Medical University; 3 Department of Statistics, University of Karachi

**Keywords:** quantile regression, cole’s lms method, who growth charts, pakistani children

## Abstract

Background

Growth charts are essential tools used by pediatricians as well as public health researchers in assessing and monitoring the well-being of pediatric populations. Development of these growth charts, especially for children above five years of age, is challenging and requires current anthropometric data and advanced statistical analysis. These growth charts are generally presented as a series of smooth centile curves. A number of modeling approaches are available for generating growth charts and applying these on national datasets is important for generating country-specific reference growth charts.

Objective

To demonstrate that quantile regression (QR) as a viable statistical approach to construct growth reference charts and to assess the applicability of the World Health Organization (WHO) 2007 growth standards to a large Pakistani population of school-going children.

Methodology

This is a secondary data analysis using anthropometric data of 9,515 students from a Pakistani survey conducted between 2007 and 2014 in four cities of Pakistan. Growth reference charts were created using QR as well as the LMS (Box-Cox transformation (L), the median (M), and the generalized coefficient of variation (S)) method and then compared with WHO 2007 growth standards.

Results

Centile values estimated by the LMS method and QR procedure had few differences. The centile values attained from QR procedure of BMI-for-age, weight-for-age, and height-for-age of Pakistani children were lower than the standard WHO 2007 centile.

Conclusion

QR should be considered as an alternative method to develop growth charts for its simplicity and lack of necessity to transform data. WHO 2007 standards are not suitable for Pakistani children.

## Introduction

Anthropometric measurements are known to be the key component of nutritional assessment of both children and adults. They are used to track the health status, nutritional competence and growth pattern in infants, children, and adolescents. Specifically, growth charts are commonly used by pediatricians and public health researchers in gauging a child’s growth progress. The most comprehensive growth charts which range from birth to 18 years of age was developed in the USA by the National Center for Health Statistics (NCHS) in 1977 which has subsequently been revised and extended by Centers for Disease Control and Prevention (CDC 2000) [1-2].

Recognizing that predominantly bottle-fed children from a single US population may not be representative of the world population, the World Health Organization (WHO) Child Growth Standards for under-five years were developed by following the growth of children from six countries (Brazil, Ghana, India, Norway, Oman, and the USA). Since it was not possible to use the same multi-country approach for school going children, the WHO Multicentre Growth Reference Study (MGRS) merged data from the 1977 NCHS growth reference to complete the 0 - 18-year-old growth references [3]. This method of merging has been questioned given that there are differences in physical development (i.e., final height achieved, age of puberty, obesity) and lifestyles between populations. In 2013, the European Society of Paediatric Gastroenterology, Hepatology, and Nutrition (ESPGHAN) Committee on Nutrition recommended that individual countries should decide the appropriateness of using the WHO growth standards for school-going children [4-8]. Similarly, for the Asian population, it has been shown that they have a higher percentage of body fat than Caucasians at the same BMI level [9]. In a systemic review that compared the WHO MGRS with data from 55 countries, Natale et al. concluded that due to wide variations in anthropometric measurements, the WHO charts are not appropriate for all countries and could put children at risk of misdiagnosis [10]. Mushtaq et al. (2012) showed that the Pakistani pediatric population significantly differ from standard references and have suggested developing national reference growth charts for Pakistani children [11]. Similarly, many other countries have also shown that WHO cut-offs are not applicable for their population [6, 7, 12]. Several countries have worked on developing their own national references for children above five years of age [13-15]. However, the development of growth charts is not an easy task. Most countries continue to rely on the WHO 2007 reference standards for nutrition and growth assessment for the above-five age group. Even with available population datasets, the use of advanced statistical techniques required to construct normalized centile curves is challenging and complex. The WHO Child Growth Charts used the Box-Cox power exponential approach (BCPE) [16] with cubic spline smoothing of curves along with power transformation of age; whereas the growth references developed by the Centers for Disease Control (CDC), Japan [13], India [14], and Turkey [15] used Cole’s LMS (Box-Cox transformation (L), the median (M), and the generalized coefficient of variation (S)) methodology for calculating percentiles and Z-scores. Apart from the LMS and BCPE method, studies in Iran [17-18] used additional statistical methods to calculate percentiles which included the Healy Rabash Yang (HRY) method, as well as the Generalized Additive Models (GAMLSS) [19]. Both LMS and BCPE methods rely on the normal theory to construct growth centiles that normalized the data by applying Box-Cox transformation. While the LMS method only transforms data once, the method used by WHO requires two transformations to achieve normality– one for the entire dataset and another on age. Two transformations create an added level of complication to the analysis.

In 1978, Koenker and Basset [20] introduced quantile regression (QR), an alternative statistical method that does not impose the assumption of normality. This regression method has the advantage of being robust when the dataset is not large and has the possibility of being a relatively easier and more flexible approach to constructing growth centiles. Several demonstrations of the usefulness of using QR for assessing the prevalence of overweight and obesity have been made using East Asian [21-22], US [23-24], and German populations [25]. In Iran, Abolfazl et al. [7, 26] used QR to develop height-for-age curves for the Iranian populations and then compared them with LMS method and WHO standards. They concluded that the WHO standards for children above five years may not be appropriate for their population and since the complex LMS method and relatively simpler QR method produced consistent results, they further suggested that QR is a flexible methodology that should be used for constructing reference growth curves.

In Pakistan, work is ongoing to provide national data but on datasets that are either not representative of the population or of the age of interest. Aziz et al. assessed nutritional status of approximately 12800 Pakistani children between the ages of 3-16 years from all four provinces of Pakistan and compared their grown centiles using CDC references [27]. The study concluded that obesity was approximately 5% and height and weight were 10-25 centile of the CDC charts. To our knowledge, in Pakistan, the QR method has only been used by Aslam et al. [28] for estimating BMI in a single population for those over the age of 14.

Our study aimed to demonstrate 1) the usefulness of QR as a method for constructing reference growth charts by comparing and contrasting it with Cole’s LMS method and WHO standard and, 2) the applicability of the WHO growth standards to a large Pakistani population of school-going age.

## Materials and methods

This is a secondary data analysis of a Pakistani national survey conducted between 2007 and 2014. The primary objective of the survey was to document the time and sequence of permanent teeth eruption in healthy Pakistani children between the ages of 4 and 15 years. The survey used systematic random sampling to assess 9,515 students from four major cities of Pakistan, namely Karachi, Larkana, Quetta, and Peshawar. The detailed methodology for collecting data from Karachi is given elsewhere [29]. The same methodology has been applied in other cities. For the purpose of this study, the anthropometric data of these children including their age, gender, height, and weight were utilized.

Statistical analysis

Cole’s LMS method and QR were applied to construct growth charts and then compared with WHO standard references. Multiple statistical software were used including SAS, SPSS, and STATA. The use of the software was determined by the extent of the software’s capabilities to handle the analysis. All the outliers for BMI were removed from the data.

QR was applied using QUANTREG procedure in SAS 9.3 software. The program for SAS is given by Chen C [30]. The higher polynomial terms of the independent variable were insignificant and thus it was decided not to transform the independent variable. This was further confirmed by cross-checking various curves in a linear regression where there the differences in the coefficient of determination between linear model and other transformed models were not large enough to warrant transformation. So, on the basis of these two procedures, it was decided to keep the model linear including the only first degree of the independent variable.

To compare the findings of QR growth charts with the procedure used by others in developing growth charts, we use the Cole’s LMS procedure in STATA 12 software. The 'colelms' command was used to calculate LMS values with effective degrees of freedom (EDF) ranging from 0 to 5. The results of all the degrees of freedom were the same and thus confirming that a transformed model is not required and a linear model can be utilized on this dataset. After calculating the LMS values, the desired percentiles were calculated using the following equation:

$$X=M(1+LSZ)^{(1/L)};L &ne;0$$

WHO standard percentiles were also estimated on this dataset using AnthroPlus software. The WHO percentiles were compared with the percentiles calculated using QR as well as the Cole’s LMS method. Since WHO standard percentiles for weight-for-age are available only for age 0 to 10 years, the comparison of three methods for weight-for-age percentiles was done only for children of age 4 to 10 years.

Ethical approval

Institutional Review Board of Dow University of Health Sciences has provided the ethical consideration for the survey (Reference no: IRB-B-147/DUHS-10). Additionally, prior to survey permission from the principal of respective schools was also obtained.

## Results

Out of 9,515 students, complete data after removing outliers and missing information was available for 8,206 students. Among these, 4,351 (53%) were males and 3,855 (47%) were females. Mean (±SD) age of the participants was 9.3 (±2.3) years. The mean (±SD) height, weight and BMI of the children were, 130.6cm (±13.7), 27.7kg (±9.6), and 15.8 kg//m2 (±3.1) respectively (Table 1). BMI-for-age, height-for-age and weight-for-age were computed for boys and girls, using QR, LMS method and WHO-percentiles.

The percentiles of WHO standard reference (available online) differed significantly from the centile charts developed using QR and LMS methods (Tables 2-7); whereas there was a small difference between the latter two methods. Most important difference observed was that, children who fell in extreme categories of WHO were in the normal ranges of both the other two methods.

According to LMS and QR method, BMI of 3.2%-3.3% of the male children was less than or equal to 3rd percentile, BMI of 82.9%-85% was >3 to ≤85th percentile and BMI of 11.7%-13.9% was above 85th percentile. However, according to WHO BMI of 23.6%, 58.2 and 18.2% of the male children was ≤3rd percentile, >3 to ≤85th percentile and >85th percentile respectively (Table 8).

For females, LMS and QR method showed BMI of 3.5%-3.6% of the children ≤3rd percentile, 84.5%-85% >3 to ≤85th percentile and 11.5%-12% >85th percentile. Whereas, according to WHO 22.3%, 67.9% and 9.7% of the children’s BMI was ≤3rd percentile, >3 to ≤85th percentile and >85th percentile respectively (Table 8).

Overall, LMS and QR method classified 3.3%-3.4% children as underweight (≤3rd percentile), 82.9%-85% normal (>3 to ≤85th percentile) and 11.6%-12.4% overweight (>85th percentile), whereas WHO classified 23% underweight, 62.8% normal and 14.2% overweight (Table 8).

**Table 1:** Descriptive statistics for demographic information

Gender	Age in years	Height (cm)				Weight (KG)			BMI (Kg/m^2^)		
		N	Mean ± SD	Min-Max	Median (IQR)	Mean ± SD	Min-Max	Median (IQR)	Mean ± SD	Min-Max	Median (IQR)
Male	4	14	116.7 ± 10.5	101 - 144	116.5 (109 - 122.3)	21.9 ± 8.6	16 - 50	20 (17 - 21.5)	15.7 ± 3.1	11.1 - 24.1	15.3 (13.8 - 17.4)
	5	100	115.4 ± 8.8	93 - 136	116 (110 - 121)	19.2 ± 4.8	7 - 36	19 (16 - 21)	14.2 ± 2.5	7.1 - 22.7	14.4 (13 - 15.7)
	6	386	115.9 ± 10.2	90 - 145	117 (109.8 - 122)	19.6 ± 5.7	7 - 55	20 (16 - 22.3)	14.4 ± 2.8	7.1 - 26.5	14.3 (12.9 - 15.8)
	7	580	120.7 ± 8.7	100 - 180	120 (115 - 125)	21.5 ± 5.4	9 - 50	20 (18 - 24)	14.6 ± 2.5	7.1 - 29.3	14.4 (13 - 15.8)
	8	625	125 ± 8.7	98 - 165	124 (119 - 130.5)	23.5 ± 5.9	13 - 60	22 (20 - 26)	14.9 ± 2.4	8.7 - 28	14.6 (13.2 - 16.2)
	9	538	131.1 ± 9	103 - 163	130 (125 - 137)	27 ± 6.8	11 - 58	25 (22 - 30)	15.5 ± 2.7	8.1 - 29.2	15.2 (13.9 - 16.6)
	10	551	135.5 ± 8.5	97 - 172	135 (129 - 141)	30.8 ± 7.9	15 - 64	30 (25 - 35)	16.6 ± 3.2	8.2 - 30	15.9 (14.6 - 18.1)
	11	624	138.4 ± 9.8	102 - 192	139 (133 - 144)	33.4 ± 8.6	16 - 82	32 (27 - 38)	17.3 ± 3.6	9.7 - 29.7	16.6 (14.7 - 19.4)
	12	477	141.5 ± 10.3	106 - 178	141 (135 - 148)	36 ± 9.1	18 - 75	35 (30 - 41)	17.8 ± 3.6	8.6 - 29.5	17.1 (15.1 - 20.2)
	13	280	147.4 ± 11.7	119 - 180	147 (139 - 155)	40.2 ± 10.7	20 - 81	38.5 (32 - 48)	18.3 ± 3.5	11.9 - 29.3	17.9 (15.6 - 20.3)
	14	139	151.1 ± 12.8	118 - 180	152 (142 - 160)	43.1 ± 12.8	19 - 89	40 (35 - 51)	18.6 ± 4.1	10.8 - 29.8	18.2 (15.6 - 20.8)
	15	37	157.9 ± 12.2	129 - 176	160 (152.5 - 165)	48.5 ± 12.1	23 - 77	48 (40.5 - 59)	19.2 ± 3.4	12.9 - 25.3	19 (16.6 - 22.1)
Female	4	16	116.3 ± 6.3	106 - 129	116.5 (111.5 - 121)	19.3 ± 3.9	15 - 28	19 (16 - 20.8)	14.3 ± 2.9	9.6 - 21.1	13.7 (13 - 14.9)
	5	113	113.5 ± 7.7	92 - 131	113 (108 - 120)	18.7 ± 4.5	9 - 35	19 (15 - 21)	14.4 ± 2.4	8.3 - 23.5	14.1 (12.9 - 16)
	6	355	113.7 ± 8.7	90 - 143	114 (108 - 119)	18.7 ± 4.9	7 - 45	19 (15 - 20)	14.3 ± 2.6	7 - 25.4	14.2 (12.9 - 15.7)
	7	501	118.8 ± 8.4	99 - 147	118 (113 - 124)	20.5 ± 5.1	8 - 49	20 (17 - 23)	14.4 ± 2.4	7.5 - 28.8	14.1 (13 - 15.7)
	8	595	124.2 ± 8.9	100 - 157	123 (119 - 129)	23 ± 5.6	10 - 50	22 (20 - 25)	14.7 ± 2.2	8 - 25.1	14.6 (13.4 - 15.8)
	9	601	129.3 ± 9.4	104 - 190	129 (123 - 134)	26.1 ± 6.4	15 - 54	25 (21 - 30)	15.4 ± 2.4	8.9 - 29.5	15.2 (13.9 - 16.6)
	10	576	134.4 ± 9.9	92 - 165	134 (128 - 141)	28.9 ± 7	15 - 61	28 (24 - 32)	15.9 ± 2.8	9.1 - 29.9	15.4 (14.1 - 17)
	11	524	138.6 ± 9.3	111 - 192	139 (132.3 - 144)	31.5 ± 7.6	15 - 60	30 (25 - 35)	16.3 ± 2.9	8.7 - 29.1	15.7 (14.3 - 17.9)
	12	319	141.9 ± 9.2	112 - 175	142 (137 - 148)	33.9 ± 8.3	19 - 65	34 (27 - 39)	16.7 ± 3.1	9.2 - 28.5	16.2 (14.3 - 18.5)
	13	167	144.8 ± 8.9	115 - 164	146 (138 - 152)	35.7 ± 8.2	20 - 70	35 (30 - 40)	16.9 ± 2.9	11.5 - 28.4	16.6 (15 - 18.3)
	14	63	145.6 ± 10.2	121 - 167	147 (138 - 152)	36.9 ± 10.6	20 - 70	35 (29 - 44)	17.2 ± 3.8	8.7 - 25.8	16.9 (14.9 - 19.5)
	15	25	145.2 ± 12.8	118 - 168	147 (134.5 - 155)	34.6 ± 10.9	18 - 62	32 (25 - 44.5)	16 ± 3.1	11.9 - 25.8	16 (13.4 - 17.6)
Overall		8206	130.6 ± 13.7	90 - 192	130 (121 - 140)	27.7 ± 9.6	7 - 89	25 (20 - 32)	15.8 ± 3.1	7 - 30	15.3 (13.8 - 17.3)

**Table 2:** BMI-for-age using quantile regression

Age in years	Age in months	Quantile regression-Percentiles (BMI kg/m^2^)										
		1%	3%	5%	15%	25%	50%	75%	85%	95%	97%	99%
For Girls												
4	48	7.6	9.3	9.9	11.8	12.4	13.5	14.6	15.5	17.1	17.7	20.0
5	60	8.1	9.6	10.3	12.0	12.6	13.8	15.0	16.0	17.8	18.5	20.9
6	72	8.5	10.0	10.6	12.3	12.9	14.1	15.5	16.5	18.5	19.2	21.8
7	84	8.9	10.4	11.0	12.5	13.2	14.5	15.9	17.0	19.2	20.0	22.7
8	96	9.3	10.8	11.3	12.8	13.5	14.8	16.3	17.4	19.8	20.8	23.6
9	108	9.8	11.2	11.7	13.1	13.7	15.1	16.7	17.9	20.5	21.5	24.5
10	120	10.2	11.6	12.1	13.3	14.0	15.4	17.2	18.4	21.2	22.3	25.3
11	132	10.6	12.0	12.4	13.6	14.3	15.8	17.6	18.9	21.9	23.1	26.2
12	144	11.1	12.4	12.8	13.9	14.5	16.1	18.0	19.4	22.6	23.8	27.1
13	156	11.5	12.8	13.2	14.1	14.8	16.5	18.5	19.8	23.2	24.6	28.0
14	168	12.0	13.2	13.5	14.4	15.1	16.8	19.0	20.4	24.0	25.5	29.0
15	180	12.3	13.5	13.9	14.6	15.4	17.1	19.3	20.8	24.6	26.1	29.8
FOR BOYS												
4	48	7.4	9.0	9.8	11.3	12.0	12.8	13.8	14.3	16.0	16.7	17.5
5	60	8.0	9.5	10.2	11.7	12.4	13.4	14.6	15.2	17.2	18.0	19.6
6	72	8.5	10.0	10.6	12.1	12.8	13.9	15.3	16.1	18.4	19.4	21.6
7	84	9.0	10.4	11.1	12.4	13.2	14.4	16.0	17.0	19.5	20.7	23.7
8	96	9.6	10.9	11.5	12.8	13.6	14.9	16.8	18.0	20.7	22.1	25.8
9	108	10.1	11.4	11.9	13.2	14.0	15.5	17.5	18.9	21.9	23.4	27.8
10	120	10.7	11.8	12.3	13.5	14.4	16.0	18.2	19.8	23.1	24.7	29.9
11	132	11.2	12.3	12.8	13.9	14.8	16.5	19.0	20.7	24.3	26.1	32.0
12	144	11.8	12.8	13.2	14.3	15.2	17.0	19.7	21.6	25.4	27.4	34.1
13	156	12.3	13.2	13.6	14.6	15.6	17.6	20.5	22.5	26.6	28.8	36.2
14	168	12.9	13.3	14.1	15.0	16.0	18.1	21.2	23.5	27.9	30.2	38.3
15	180	13.2	14.1	14.4	15.3	16.3	18.5	21.8	24.1	28.7	31.1	39.8

**Table 3:** Height-for-age using quantile regression

Age in years	Age in months	Quantile regression-Percentiles (Height in cm)										
		1%	3%	5%	15%	25%	50%	75%	85%	95%	97%	99%
	FOR GIRLS											
4	48	91.4	93.2	94.0	98.5	101.3	106.0	111.4	115.8	123.8	127.0	132.0
5	60	94.6	96.8	98.0	102.8	105.7	110.5	116.0	120.2	128.0	131.0	136.0
6	72	97.7	100.4	102.0	107.0	110.0	115.0	120.6	124.6	132.2	135.0	140.0
7	84	100.9	104.0	106.0	111.3	114.4	119.5	125.2	129.0	136.4	139.0	144.0
8	96	104.0	107.6	110.0	115.5	118.7	124.0	129.8	133.4	140.5	143.0	148.0
9	108	107.2	111.2	114.0	119.8	123.0	128.5	134.4	137.8	144.7	147.0	152.0
10	120	110.3	114.8	118.0	124.0	127.4	133.1	139.1	142.2	148.9	151.0	156.0
11	132	113.5	118.5	122.1	128.3	131.7	137.6	143.7	146.7	153.1	155.1	160.1
12	144	116.7	122.1	126.1	132.6	136.1	142.1	148.3	151.1	157.3	159.1	164.1
13	156	119.9	125.8	130.2	137.0	140.6	146.7	153.0	155.6	161.6	163.2	168.2
14	168	123.3	129.7	134.6	141.6	145.3	151.7	158.1	160.5	166.1	167.6	172.6
15	180	126.0	132.8	138.0	145.3	149.0	155.5	162.0	164.2	169.7	171.0	176.0
FOR BOYS												
4	48	93.3	94.2	95.7	100.0	104.0	108.0	113.8	117.0	120.0	124.9	130.3
5	60	95.7	97.6	99.4	104.0	108.0	112.4	118.2	121.6	125.1	129.6	135.1
6	72	98.0	101.0	103.0	108.0	112.0	116.7	122.6	126.0	130.0	134.3	139.8
7	84	100.4	104.4	106.7	112.0	116.0	121.0	127.0	130.5	135.0	139.0	144.5
8	96	102.7	107.8	110.4	116.0	120.0	125.4	131.4	135.0	140.0	143.7	149.3
9	108	105.0	111.2	114.0	120.0	124.0	129.7	135.8	139.5	145.1	148.5	154.0
10	120	107.4	114.6	117.7	124.0	128.0	134.1	140.3	144.1	150.1	153.2	158.8
11	132	109.7	118.0	121.4	128.0	132.0	138.4	144.7	148.6	155.1	157.9	163.6
12	144	112.0	121.5	125.1	132.1	136.1	142.7	149.1	153.1	160.1	162.7	168.3
13	156	114.4	124.9	128.8	136.1	140.1	147.1	153.5	157.6	165.2	167.4	173.2
14	168	116.8	128.4	132.6	140.3	144.3	151.6	158.1	162.3	170.4	172.3	178.1
15	180	118.5	130.9	135.2	143.1	147.1	154.7	161.3	165.5	173.9	175.7	181.5

**Table 4:** Weight-for-age using quantile regression

Age in years	Age in months	Quantile regression-Percentiles (Weight in Kg)										
		1%	3%	5%	15%	25%	50%	75%	85%	95%	97%	99%
FOR GIRLS												
4	48	6.5	8.4	9.2	11.3	12.0	13.2	16.0	18.0	22.0	22.5	25.8
5	60	8.0	10.0	10.8	13.0	14.0	15.6	18.7	21.0	25.4	26.3	30.0
6	72	9.5	11.6	12.4	14.8	16.0	18.0	21.4	24.0	28.7	30.0	34.2
7	84	11.0	13.2	14.0	16.5	18.0	20.4	24.2	27.0	32.0	33.8	38.4
8	96	12.5	14.7	15.6	18.3	20.0	22.8	26.9	30.0	35.4	37.5	42.5
9	108	14.0	16.3	17.2	20.0	22.0	25.2	29.6	33.0	38.7	41.3	46.7
10	120	15.5	17.9	18.8	21.8	24.0	27.6	32.3	36.0	42.0	45.0	50.9
11	132	17.0	19.5	20.4	23.5	26.0	30.0	35.0	39.0	45.4	48.8	55.1
12	144	18.5	21.0	22.0	25.3	28.1	32.5	37.8	42.1	48.8	52.6	59.3
13	156	20.1	22.7	23.7	27.1	30.1	34.9	40.6	45.2	52.2	56.4	63.6
14	168	21.7	24.4	25.4	29.0	32.3	37.6	43.6	48.5	55.8	60.6	68.1
15	180	23.0	25.7	26.8	30.5	34.0	39.6	45.9	51.0	58.7	63.8	71.7
FOR BOYS												
4	48	5.0	7.0	8.0	11.0	10.0	12.8	15.0	16.7	20.3	21.0	25.0
5	60	7.0	9.0	10.0	13.0	12.5	15.5	18.4	20.4	24.5	25.8	30.1
6	72	9.0	11.0	12.0	15.0	15.0	18.3	21.7	24.0	28.8	30.5	35.0
7	84	11.0	13.0	14.0	17.0	17.5	21.0	25.0	27.7	33.0	35.3	40.0
8	96	13.0	15.0	16.0	19.0	20.0	23.8	28.4	31.4	37.3	40.0	45.0
9	108	15.0	17.0	18.0	21.0	22.5	26.5	31.7	35.0	41.5	44.8	50.1
10	120	17.0	19.0	20.0	23.0	25.0	29.3	35.0	38.7	45.8	49.6	55.1
11	132	19.0	21.0	22.0	25.0	27.5	32.0	38.4	42.4	50.1	54.3	60.1
12	144	21.0	23.0	24.0	27.0	30.0	34.8	41.7	46.1	54.3	59.1	65.1
13	156	23.1	25.1	26.1	29.1	32.6	37.6	45.1	49.8	58.6	63.9	70.2
14	168	25.1	27.1	28.1	31.1	35.2	40.5	48.6	53.6	63.1	68.8	75.4
15	180	26.6	28.6	29.6	32.6	37.0	42.4	51.0	56.2	66.1	72.2	78.9

**Table 5:** BMI-for-age using the LMS method

Age in years	Age in months	Cole’s LMS-Percentiles (BMI kg/m^2^)										
		1%	3%	5%	15%	25%	50%	75%	85%	95%	97%	99%
For Girls												
4	48	9.2	9.9	10.3	11.5	12.2	13.9	16	17.3	19.8	21	23.4
5	60	9	9.9	10.5	11.9	12.7	14.4	16.1	17	18.6	19.2	20.4
6	72	8.6	9.6	10.2	11.6	12.5	14.3	16.1	17.1	18.8	19.5	20.8
7	84	9.3	10.2	10.6	11.9	12.7	14.3	15.9	16.9	18.6	19.2	20.6
8	96	10.5	11.1	11.5	12.5	13.2	14.5	16.1	17	18.7	19.4	20.9
9	108	11.2	11.8	12.2	13.2	13.8	15.2	16.8	17.9	19.9	20.8	22.7
10	120	11.3	11.9	12.3	13.3	14	15.5	17.4	18.6	21.1	22.2	24.6
11	132	11.1	11.8	12.2	13.4	14.2	15.9	17.9	19.2	21.5	22.6	24.7
12	144	11.3	12.1	12.5	13.7	14.5	16.3	18.4	19.8	22.4	23.6	26.2
13	156	12.1	12.8	13.2	14.3	15	16.6	18.5	19.8	22.3	23.5	26.1
14	168	9.5	10.7	11.4	13.4	14.6	17.1	19.7	21.2	23.9	24.9	27.1
15	180	10.9	11.6	12	13.1	13.9	15.6	17.7	19.1	21.9	23.2	26.1
FOR BOYS												
4	48	10.6	11.3	11.7	12.9	13.6	15.3	17.5	18.9	21.8	23.2	26.3
5	60	8	9.3	10	11.7	12.7	14.5	16.2	17.1	18.6	19.2	20.2
6	72	8.3	9.4	10	11.5	12.5	14.4	16.3	17.4	19.2	20	21.4
7	84	9.6	10.4	10.9	12.1	12.9	14.4	16.1	17.1	18.9	19.6	21.1
8	96	10.6	11.2	11.6	12.6	13.2	14.6	16.3	17.3	19.3	20.2	22.1
9	108	11	11.6	12	13	13.7	15.2	17	18.1	20.3	21.3	23.3
10	120	11.3	12	12.4	13.6	14.4	16.2	18.4	19.8	22.7	24	26.9
11	132	11.3	12.1	12.6	13.9	14.8	16.8	19.3	21	24.3	25.8	29.1
12	144	11.4	12.3	12.8	14.3	15.3	17.4	19.9	21.4	24.4	25.6	28.3
13	156	12.3	13.1	13.6	14.9	15.9	17.9	20.3	21.9	25	26.4	29.5
14	168	11.1	12.2	12.8	14.5	15.7	18.1	21.1	22.8	26.3	27.8	30.8
15	180	12.3	13.4	14	15.7	16.8	19	21.4	22.7	25.2	26.3	28.3

**Table 6:** Height-for-age using the LMS method

Age in years	Age in months	Cole’s LMS-Percentiles (Height in cm)										
		1%	3%	5%	15%	25%	50%	75%	85%	95%	97%	99%
	FOR GIRLS											
4	48	101.8	104.5	106	109.7	112	116.2	120.5	122.9	126.8	128.4	131.3
5	60	95.7	99.1	100.9	105.6	108.4	113.6	118.8	121.6	126.3	128.2	131.7
6	72	93.8	97.5	99.5	104.7	107.8	113.6	119.6	122.8	128.3	130.5	134.5
7	84	101.2	104.2	105.9	110.3	113	118.4	124.2	127.5	133.3	135.7	140.3
8	96	107.1	109.8	111.4	115.6	118.3	123.8	129.9	133.6	140.3	143.2	149
9	108	111.2	114.1	115.7	120.2	123.1	128.8	135.3	139.1	146.1	149.1	155
10	120	110.7	115.5	118.1	124.5	128.2	135	141.7	145.2	150.9	153.2	157.3
11	132	117.9	121.7	123.7	129	132.2	138.3	144.7	148.2	154.2	156.6	161.1
12	144	118.3	124	126.8	133.5	137.2	143.7	149.7	152.7	157.6	159.4	162.8
13	156	121.9	127.4	130.1	136.7	140.3	146.6	152.4	155.4	160	161.8	165
14	168	118.8	125.5	128.8	136.6	140.8	148	154.6	157.9	163.2	165.1	168.7
15	180	110.5	119.3	123.5	133.5	138.9	148.1	156.5	160.7	167.4	169.9	174.4
FOR BOYS												
4	48	99.9	102.4	103.9	108	110.8	116.7	124.1	128.8	138.6	143.3	153.9
5	60	94	98.5	100.8	106.6	110	116.1	121.9	125	130.1	132	135.5
6	72	92	96.7	99.2	105.5	109.3	116.2	123.1	126.8	132.9	135.3	139.8
7	84	103.9	106.6	108.1	112.2	114.9	120.2	126.2	129.8	136.3	139	144.6
8	96	106.7	109.8	111.5	116.1	118.9	124.6	130.6	134.1	140.1	142.6	147.5
9	108	111.8	115.2	117	121.9	124.9	130.8	136.9	140.4	146.4	148.8	153.5
10	120	115.6	119.5	121.5	126.8	129.9	135.7	141.5	144.6	149.8	151.8	155.5
11	132	116.2	120.3	122.6	128.4	131.8	138.4	145	148.6	154.7	157.1	161.6
12	144	118.6	122.8	125	130.9	134.5	141.3	148.4	152.3	158.9	161.6	166.6
13	156	122.1	126.5	128.9	135.3	139.3	146.9	155	159.4	167.2	170.4	176.4
14	168	119.8	126.5	130	138.6	143.5	152.4	160.9	165.3	172.6	175.3	180.4
15	180	113.4	131.4	137.9	150.7	156.7	165.8	173.3	176.9	182.2	184.2	187.6

**Table 7:** Weight-for-age using the LMS method

Age in years	Age in months	Cole’s LMS-Percentiles (Weight in Kg)										
		1%	3%	5%	15%	25%	50%	75%	85%	95%	97%	99%
FOR GIRLS												
4	48	12.7	13.7	14.2	15.6	16.4	18	19.6	20.5	22	22.6	23.8
5	60	9	10.8	11.7	14.2	15.7	18.7	21.6	23.3	26.1	27.2	29.2
6	72	9.6	11.3	12.2	14.5	15.9	18.4	21	22.4	24.7	25.6	27.3
7	84	12	13.3	14	16	17.3	19.9	22.8	24.5	27.5	28.7	31.2
8	96	13.8	15.2	15.9	17.9	19.2	21.8	24.5	26.1	28.9	30	32.3
9	108	15.6	17	17.8	20	21.4	24.6	28.3	30.6	35	36.9	40.9
10	120	17	18.6	19.6	22.2	23.9	27.4	31.5	33.8	38.2	40.1	43.8
11	132	17.8	19.8	20.9	24	26	30.2	34.9	37.7	42.9	45.1	49.5
12	144	19.2	21.3	22.5	26	28.3	33	38.5	41.8	47.9	50.5	55.8
13	156	19.4	22.2	23.7	27.8	30.3	35.2	40.2	43	47.9	49.8	53.5
14	168	17.3	20.2	21.8	26.4	29.4	35.6	42.6	46.7	54.2	57.3	63.4
15	180	14.5	17.4	19.1	23.7	26.8	32.9	39.7	43.6	50.6	53.5	59.1
FOR BOYS												
4	48	15.2	15.9	16.4	17.8	18.8	21.4	25.4	28.9	40.9	52.8	65.2
5	60	8.2	10.2	11.3	14.2	16	19.4	22.8	24.7	27.9	29.2	31.6
6	72	8.8	10.4	11.3	13.8	15.5	19.1	23.1	25.5	29.9	31.7	35.4
7	84	12	13.3	14.1	16.2	17.7	20.8	24.5	26.8	31.2	33.2	37.2
8	96	14.3	15.5	16.1	18.1	19.5	22.6	26.6	29.2	34.6	37.1	42.8
9	108	15.7	17.2	18	20.5	22.2	26	30.6	33.6	39.5	42.1	47.9
10	120	17.8	19.5	20.5	23.3	25.3	29.6	35	38.5	45.5	48.7	55.6
11	132	19.3	21.1	22.1	25.2	27.3	32	38	41.9	49.8	53.4	61.5
12	144	20.8	22.7	23.9	27.2	29.5	34.6	40.9	45	53.3	57	65.2
13	156	21.8	24.2	25.6	29.7	32.5	38.6	46.1	50.9	60.4	64.7	73.8
14	168	21.1	23.9	25.6	30.5	33.9	41.2	50.2	55.8	66.9	71.7	81.9
15	180	21	26.1	28.8	36.1	40.5	48.9	57.5	62.2	70.2	73.3	79.3

**Table 8:** Prevalence of malnourished children using three methods

Percentiles	BMI; n(%)			Height; n(%)			Weight; n(%) (For age 4-10 years)		
	LMS^a^	WHO^b^	QR^c^	LMS^a^	WHO^b^	QR^c^	LMS^a^	WHO^b^	QR^c^
Male									
<=3	143 (3.3)	1027 (23.6)^a,c^	140 (3.2)	132 (3)	854 (19.6)^a,c^	113 (2.6)	98 (3.5)	588 (21)^a,c^	102 (3.7)
>3 to 85	3697 (85)^b,c^	2532 (58.2)^c^	3605 (82.9)	3661 (84.1)^b^	2782 (63.9)	3606 (82.9)^b^	2315 (82.9)^c^	1745 (62.5)	2319 (83)^c^
>85	511 (11.7)	792 (18.2)^a,c^	606 (13.9)^a^	558 (12.8)	715 (16.4)^a,c^	632 (14.5)	381 (13.6)	461 (16.5)^a,c^	373 (13.4)
Total	4351 (100)	4351 (100)	4351 (100)	4351 (100)	4351 (100)	4351 (100)	2794 (100)	2794 (100)	2794 (100)
Female									
<=3	137 (3.6)	861 (22.3)^a,b^	124 (3.2)	136 (3.5)	835 (21.7)^a,c^	111 (2.9)	115 (4.2)	654 (23.7)^a,b^	80 (2.9)
>3 to 85	3275 (85)^b^	2619 (67.9)	3200 (83)^b^	3256 (84.5)^b^	2530 (65.6)	3198 (83)^b^	2282 (82.8)^b^	1753 (63.6)	2353 (85.3)^b^
>85	443 (11.5)^b^	375 (9.7)	531 (13.8)^b^	463 (12)	490 (12.7)	546 (14.2)^a^	360 (13.1)^b^	350 (12.7)	324 (11.8)^a,b^
Total	3855 (100)	3855 (100)	3855 (100)	3855 (100)	3855 (100)	3855 (100)	2757 (100)	2757 (100)	2757 (100)
Overall									
<=3	280 (3.4)	1888 (23)^a,c^	264 (3.2)	268 (3.3)	1689 (20.6)^a,c^	224 (2.7)	213 (3.8)	1242 (22.4)^a,c^	182 (3.3)
>3 to 85	6972 (85)^b,c^	5151 (62.8)	6805 (82.9)	6917 (84.3)^b^	5312 (64.7)	6804 (82.9)^b^	4597 (82.8)^b^	3498 (63)	4672 (84.2)^b^
>85	954 (11.6)	1167 (14.2)^a^	1137 (13.9)	1021 (12.4)	1205 (14.7^a^)	1178 (14.4)^a^	741 (13.3)	811 (14.6)^a,c^	697 (12.6)
Total	8206 (100)	8206 (100)	8206 (100)	8206 (100)	8206 (100)	8206 (100)	5551 (100)	5551 (100)	5551 (100)
Results are based on two-sided tests with significance level .05. For each significant pair, the key of the category (a,b,c) with the smaller column proportion appears under the category with the larger column proportion									

## Discussion

Our study is the first published study from Pakistan that has created a Pakistani growth chart references for school-going children, between the ages of 4-15 years, using QR, a statistically robust procedure that does not require complex transformations. We found centile values of BMI-for-age, weight-for-age, and height-for-age of Pakistani children was lower than the standard WHO 2007 centile values, using both the LMS method to establish growth chart references as well as the QR procedure. We further showed that WHO 2007 standards for school-going children were inappropriate for Pakistani population.

Our analysis demonstrated that using WHO 2007 standard growth references for our population may place children at a risk of misdiagnosis; inferring that Pakistani researchers and pediatricians should not use WHO 2007 growth charts as references to compare growth. Specifically, we found that the centile values for BMI-for-age, height-for-age, and weight-for-age of Pakistani children are below the WHO 2007 standard references implying that if researchers and pediatricians use the WHO standard references to estimate nutritional status, they may overestimate the prevalence of underweight in this age-group and pediatricians may incorrectly identify a child as being stunted, underweight, or overweight.

Even though our study was not on affluent children, an Indian study on affluent children [6] also reported similar findings to our study. Khadilkar et al. concluded that that average Z-scores for height-for-age, weight-for-age, BMI-for-age and weight-for-height of Indian preschool children were below the WHO 2007 standard median. This further highlight that using the WHO 2007 standards may not be accurate for the South Asian population, possibly due to genetic and environmental differences between our population and that of the WHO reference population. Even the European Commission on nutrition could not recommend using the WHO 2007 standards for school-going children and recommended that each European nation self-determine the usefulness of adopting the WHO standards for use in their country [8].

In Pakistan, Aziz et al. conducted a large study on school-going children and presented data to policy makers for consideration as the national reference. Whereas our study population and that of Aziz et al. [27] were similar in age groups, our study has the strength of establishing independent growth references for Pakistani children over the age of five using appropriate and acceptable statistical procedures and not depending on the CDC growth charts. Furthermore, our study had almost an equal distribution of boys and girls whereas the study of Aziz et al. stated their limitation as being a disparity in the number of boys and girls.

## Conclusions

This secondary data analysis showed that the anthropometric measurements of Pakistani children, calculated using WHO 2007 standard references, are not suitable for our population. It further highlighted that QR can be successfully used as an alternative method to develop reference growth charts. This study has contributed to the growing literature that each country needs to build its own reference growth charts and not rely on growth charts that are not representative of its own pediatric population. We recommend pooling of multiple Pakistani datasets and creating a Pakistani reference growth chart.
